# The clinical course of schizophrenia in women and men—a nation-wide cohort study

**DOI:** 10.1038/s41537-020-0102-z

**Published:** 2020-05-01

**Authors:** Iris E. Sommer, Jari Tiihonen, Anouk van Mourik, Antti Tanskanen, Heidi Taipale

**Affiliations:** 1 Rijksuniversiteit Groningen (RUG), University Medical Center Groningen (UMCG), Department of Biomedical Sciences of Cells and Systems, Groningen, Netherlands; 2 Department of Clinical Neuroscience, Karolinska Institutet, Stockholm, Sweden; 3 Department of Forensic Psychiatry, University of Eastern Finland, Niuvanniemi Hospital, Kuopio, Finland; 4 Center for Psychiatry Research, Stockholm City Council, Stockholm, Sweden; 5 School of Pharmacy, University of Eastern Finland, Kuopio, Finland

**Keywords:** Psychosis, Schizophrenia

## Abstract

Gender differences in schizophrenia have been reported in different aspect of the course of disease and may urge special clinical interventions for female patients. Current literature provides insufficient information to design guidelines for treating women with schizophrenia. We aim to quantify the clinical course of schizophrenia in men and women on premorbid hospitalizations and prescription drugs, age at diagnosis, pharmacological treatment, comorbidity, number of re-hospitalizations, and mortality. Our nationwide cohort study included all patients admitted for the first time to hospital during 2000–2014 for schizophrenia or schizo-affective disorder in Finland. Gender differences were compared with logistic regression, by calculating incidence rates, and mortality was assessed with Cox proportional hazard model. We included 7142 women and 9006 men with schizophrenia/schizo-affective disorder and found that both women (71%) and men (70%) had often been hospitalized for another psychiatric disorder in the 5 years before diagnosis. In women, the last psychiatric hospitalization before schizophrenia/schizo-affective diagnosis was often for mood disorders (62%, OR 2.56, 95% CI 2.28–2.87). Men were diagnosed earlier (mean 34.4 [SD12.6] vs. 38.2 [SD 13.8]) with peak incidence around 22, while incidence in women declining only slowly between age 18 and 65. During ten years follow-up, 69.5% of both genders needed at least one re-hospitalization, with slightly more hospitalizations in women. Women were less often prescribed clozapine or long-acting antipsychotics. Mortality was lower in women (HR = 0.54, 95% CI 0.50–0.60), with fewer suicide and cardiovascular deaths, but more cancer deaths. These results suggest a diagnostic delay for women, which might be shortened by screening women aged 20–65 participating in affective disorder programs. As number of hospitalizations is not lower for women, clinicians should take care not to undertreat women with schizophrenia.

## Introduction

Gender differences in schizophrenia affect many domains, including premorbid trajectory, incidence, symptoms, comorbidity, outcome, and mortality^1–3^. The most consistently reported gender difference is the higher age at onset in women^2,3^. After diagnosis, the course of disease may also vary between the genders, but literature is less consistent on this aspect^4–9^. Symptoms of schizophrenia may be gender dimorphic too, with approximately half of the studies showing more depressive symptoms in women and more negative symptoms in men, and the other half showing no difference^5^. Women may have the additional benefit of responding better to treatment, yet this benefit appears to dissipate with advancing age^4^. Comorbid substance abuse is higher in men, which applies to cannabis, cocaine, hallucinogens and alcohol, while depression is more common in women^6,7^. High prevalence of substance abuse in men with schizophrenia may contribute both to earlier onset and to more severe course in men as compared to women. Mortality has been reported less frequently, with some studies indicating similar rates in men and women^8–10^, but a recent meta-analysis showing higher mortality in men^9^. In sum, gender differences are present in schizophrenia, with most consistent findings for later onset in women and less consistent data on a possibly better course in and lower mortality in women. This raises the question: if schizophrenia expresses itself different in women than in men, should we develop different guidelines for (early) diagnosis and treatment of women? We could envision different expectations about prognosis, specific treatment for comorbidities, and perhaps milder pharmacological treatment. For both men and women with schizophrenia, duration of untreated psychosis (DUP) is an important predictor of outcome^10^. Therefore, early detection is another domain that might benefit from a gendered-approach^5^. As the presentation of schizophrenia in women may be less typical than in men, women run the risk for diagnostic delay, potentially reducing their chances for good outcome. Another potential difference between the genders could be the better response to medication in women, which may result in lower relapse rates. Current literature provides insufficient detailed data on the complete course of schizophrenia in men and women to make clear guideline recommendation for diagnosis and treatment for women. What is needed for such guidelines is longitudinal data from a large group of men and women with schizophrenia to assess early trajectory, age and type of diagnosis, morbidity and comorbidity but also mortality in the same patients. In this study, we aim to describe the clinical course of schizophrenia in both genders, including hospitalizations and psychiatric medication use in the premorbid period, age at diagnosis, pharmacological treatment, comorbidity, number of re-hospitalizations, and mortality in the same persons. We use the Finnish registers, which (together with the Danish registers) hold the most complete and longest follow-up data. As affective symptoms are very common in women, we include patients with either schizophrenia or schizo-affective disorders. Since schizo-affective disorder may represents ‘the more female form of schizophrenia’, restricting inclusion to only schizophrenia would introduce selection bias, especially for the women.

## Results

We included 16,148 patients with a schizophrenia-spectrum disorder (SSD). Of these, 7142 were women and 9006 men (ratio 1:1.3). SSD consisted of two diagnoses: schizophrenia and schizo-affective disorder. Schizoaffective disorder was diagnosed for 31.2% (*N* = 2231) of women and 17.5% (*N* = 1577) of men.

### Five years before diagnosis of SSD

Most women (71.2%) and men (70.1%) had been hospitalized for another psychiatric disorder (psychotic and/or other) before the first hospitalization for SSD. 47.8% (*N* = 3412) of women had been hospitalized for another psychotic disorder, and this resulted into a median of 56 hospital days (IQR 24–115), while the median number of hospital days for any psychiatric diagnosis (from 70.5% of women, *N* = 5038) was 81 (IQR 34–178). In men, the median number of hospital days for another psychotic disorder before SSD was 53 (IQR 21–109) for 48.5% of men (*N* = 4368), with corresponding median of hospital days due to any psychiatric diagnosis of 65 (IQR 25–146, 69.4% of men, (*N* = 6252).

Significant differences occurred in type of diagnoses in the trajectory before SSD, with more frequent mood disorders (with and without mania), anxiety disorders, eating disorders, post-traumatic stress disorder, dissociative disorder, personality disorder, suicide attempts, and self-harm in women (Table 1). Men had more often substance disorder and more autism spectrum disorders. The largest gender differences were found for eating disorder (ten times more prevalent for women) and substance abuse, for which women had less than half the risk as men (Table 1).

**Table 1:** The prevalence of psychiatric diagnoses before and after a schizophrenia-spectrum diagnosis (SDD) was made, for men (*n* = 9006) and women (*n* = 7142).

Psychiatric comorbidities	Before first hospitalization with diagnosis SDD				After first hospitalization with diagnosis SDD			
	Women (%)	Men (%)	*p*-value	OR (95% CI) for women	Women (%)	Men (%)	*p*-value	OR (95% CI) for women
Developmental disorders (autism spectrum)	1.7	2.4	0.0020	0.70 (0.56–0.88)	0.6	1.2	0.0002	0.51 (0.35–0.73)
Developmental disorders (ADHD spectrum)	3.6	4.3	0.0222	0.83 (0.71–0.97)	0.5	0.8	0.0167	0.62 (0.42–0.92)
Eating disorders	2.9	0.30	<0.0001	10.19 (6.77–15.33)	1.5	0.1	<0.0001	10.5 (5.9–18.66)
PTSD (Post-Traumatic Stress Disorder)	0.9	0.4	0.0010	1.93 (1.30–2.89)	0.5	0.2	0.0001	3.0 (1.67–5.39)
OCD (Obsessive-Compulsive Disorder)	1.5	1.6	0.4855	0.92 (0.71–1.18)	1.1	0.9	0.1877	1.23 (0.9–1.68)
Suicide attempt or self-harm	11.7	8.6	<0.0001	1.42 (1.28–1.57)	8.2	5.9	<0.0001	1.4 (1.24–1.59)
Anxiety disorders	10.9	9.2	0.0007	1.20 (1.08–1.33)	5.0	3.6	<0.0001	1.4 (1.21–1.66)
Personality disorders	17.9	15.6	0.0001	1.18 (1.08–1.28)	9.1	6.8	<0.0001	1.37 (1.22–1.54)
Mood disorders without mania	30.7	21.1	<0.0001	1.66 (1.54–1.78)	9.8	5.8	<0.0001	1.78 (1.58–2.0)
Dissociative, somatoform and neurasthenic disorders	2.1	1.1	<0.0001	1.90 (1.47–2.44)	1.2	0.4	<0.0001	3.25 (2.19–4.84)
Substance-use related disorders	13.2	26.0	<0.0001	0.43 (0.40–0.47)	12.0	22.2	<0.0001	0.48 (0.44–0.52)
Other psychotic disorders	54.7	53.8	0.2199	1.04 (0.98–1.11)				
Sleeping disorders	1.0	1.1	0.4468	0.89 (0.66–1.21)	0.6	0.6	0.7893	1.06 (0.71–1.56)
Mood disorders with mania	10.6	6.7	<0.0001	1.66 (1.48–1.85)	5.5	3.5	<0.0001	1.63 (1.4–1.9)

*P*-values represent the difference in gender for the prevalence of diagnoses.

**Fig. 1:**
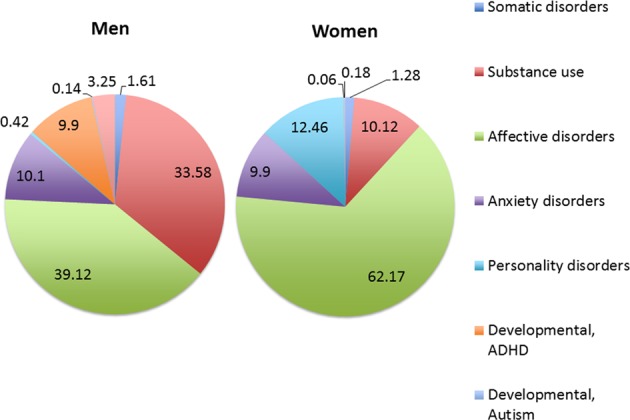
Last diagnosis preceding first hospitalisation for schizophrenia-spectrum disorder. Last recorded non-psychotic diagnosis for women (right) (*n*=2263) and men (left) (*n*=2615) before hospitalization for SSD, for those who had at least 1 prior hospitalization.

Gender differences in the age of first hospitalization were most pronounced for eating disorders, occurring much earlier in women (mean age in women 21.6, SD 7.9, against 29.4, SD 12.8, in men, *p* < 0.0001). For women, mood disorder (with and without mania combined) was the most common last diagnosis preceding SSD in 62% (against only 39% in men) (Fig. 1). For men, 34% was hospitalized for substance disorder preceding the SSD hospitalization (against only 10% for women).

**Fig. 2:**
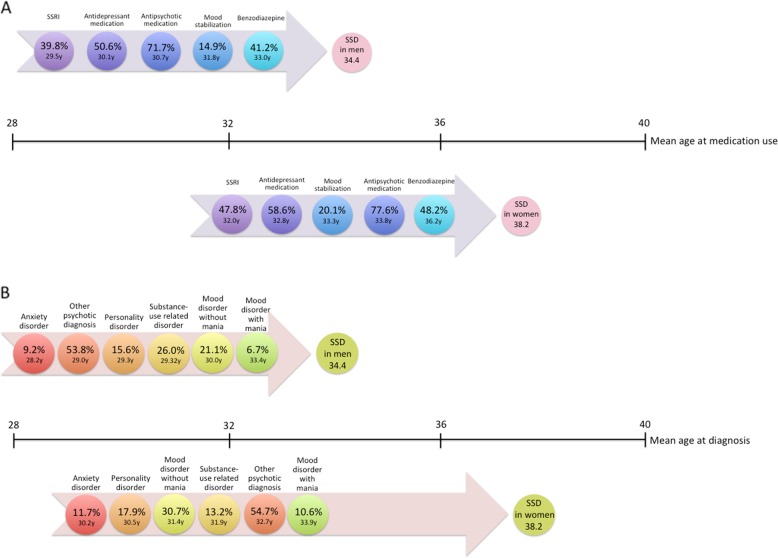
Medication use and diagnosis preceding first hospitalisation for schizophrenia-spectrum disorder. Prevalence and mean age for (**a**) medication use before first hospitalization for schizophrenia-spectrum diagnosis (SSD) and for (**b**) other psychiatric disorders, by gender (men *n* = 9006, women *n* = 7142). SSRI: Serotonin reuptake inhibitors.

The vast majority of both women (78%) and men (72%) already used antipsychotic medication before SSD diagnoses. Antidepressants were also used by more than half of women (59%) and men (51%). Benzodiazepine use was high amongst both genders (48% for women and 41% for men), but use of benzodiazepines was started relatively late in the premorbid trajectory. Overall, men started earlier with the use of psychiatric medications than women, but women used them more frequently. The order of medication use in both sexes started with SSRIs, followed by other antidepressant medication and ended with benzodiazepines. Women started earlier with mood stabilization, while men started earlier with antipsychotics. Figure 2 shows the medication use (upper arrows) and previous diagnoses (lower arrows) in the premorbid trajectory for women and men.

### Age at diagnosis

**Fig. 3:**
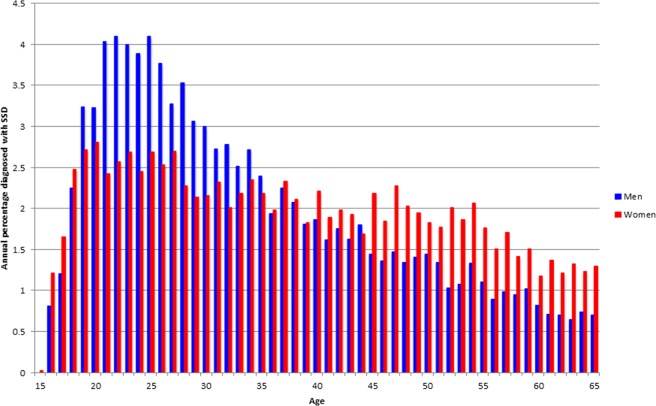
Age at diagnosis of schizophrenia-spectrum disorder. Age of first recorded schizophrenia-spectrum diagnosis for men (*n*=9006) and women (*n* = 7142).

Men were on average diagnosed earlier (mean 34.4, SD 12.6, median 31, IQR 24–43) than women (mean 38.2, SD 13.8, median 37, IQR 26–50). In women, but not in men, there appears to be a gap between the last hospitalization for another psychiatric disorder and the first hospitalization for SSD of some 4 years (Fig. 1b), during which half the women start with benzodiazepines (Fig. 2a). While men showed a peak around the age of 22, with a distribution skewed to the right, women showed a more plateau-like distribution between the ages of 18 and 65, with slightly higher values between the ages of 18 and 35 and only slowly decreasing incidence (Fig. 3).

### Re-hospitalizations after SSD diagnoses

During the follow-up time of up to 10 years (mean 7.9, SD 2.8 for men and 8.2, SD 2.5 for women), 69.5% of men and women had a psychiatric re-hospitalization. Women had a somewhat higher rate of psychiatric re-hospitalizations (incidence rate per 10 person-years 5.18, 95% CI 5.16–5.20) then men (5.12, CI 5.10–5.13).

Per 10 person-years, women had a incidence rate of hospitalizations for psychotic disorders of 4.13 (CI 4.11–4.15) and for other psychiatric disorders of 5.18 (CI 5.16–5.20). In men, numbers were quite similar for psychotic hospitalizations (4.16 CI 4.15–4.18), with slightly lower for other psychiatric disorders (5.12 CI 5.10–5.13). Mean duration of hospitalizations were similar for both genders.

Figure 2 of the Supplementary material shows comorbid psychiatric hospitalizations after SSD for women and men, with more hospitalizations for substance-use disorders in men, but higher frequency of hospitalizations for all other comorbidities in women. Hospitalization for suicide attempts and self-harm was also significantly more prevalent among women (8.2%) than among men (5.9%).

### Pharmacotherapy

Medication use during the first 5 years after the first hospitalization for SSD is displayed in Table 2, showing that 96.6% of women and 94.4% of men used antipsychotic medication. Men more often used clozapine, olanzapine and long-acting antipsychotics, while women more often used quetiapine and aripiprazole. Odds of using antidepressant and mood stabilizing medication was about 40% higher in women. The use of benzodiazepine and zopiclon/zolpidem was significantly higher in women.

**Table 2:** Prevalence of psychiatric medication use during the five years before, 1 year after and fifth year after the first hospitalization with schizophrenia-spectrum diagnosis (SSD) for women (*n* = 7142) and men (*n* = 9006).

	Medication use during 5 years before SSD				Medication use during first five years after SSD			
	Women (%)	Men (%)	*p*-value	OR (95% CI) for women	Women (%)	Men (%)	*p*-value	OR (95% CI) for women
Antipsychotics	77.6	71.7	<0.0001	1.37 (1.28–1.47)	96.6	94.4	<0.0001	1.7 (1.46–2.0)
Antidepressants	58.6	50.6	<0.0001	1.38 (1.3–1.47)	60.0	52.5	<0.0001	1.36 (1.27–1.45)
SSRIs	47.8	39.8	<0.0001	1.38 (1.3–1.47)	45.7	37.7	<0.0001	1.39 (1.31–1.48)
Other antidepressants	28.8	26.2	<0.0001	1.2 (1.12–1.29)	33.3	29.5	<0.0001	1.2 (1.12–1.28)
Benzodiazepines	48.2	41.2	<0.0001	1.3 (1.21–1.38)	53.1	45.2	<0.0001	1.37 (1.29–1.46)
Mood stabilizers	20.1	14.9	<0.0001	1.44 (1.33–1.56)	33.1	26.1	<0.0001	1.4 (1.31–1.5)
Zopiclone and zolpidem	22.9	17.6	<0.0001	1.39 (1.28–1.5)	26.2	19.8	<0.0001	1.43 (1.33–1.54)
ADHD medication	0.4	0.9	<0.0001	0.37 (0.24–0.58)	0.3	0.8	<0.0001	0.35 (0.21–0.56)
*Specific antipsychotics*								
Clozapine	5.0	4.1	0.0063	1.23 (1.06–1.43)	26.9	31.4	<0.0001	0.8 (0.75–0.86)
Olanzapine	27.8	29.0	0.1017	0.94 (0.88–1.0)	43.5	45.8	0.0027	0.91 (0.85–0.97)
Quetiapine	29.3	21.9	<0.0001	1.48 (1.38–1.59)	45.1	35.7	<0.0001	1.48 (1.39–1.58)
Risperidone	33.6	32.7	0.2269	1.04 (0.98–1.1)	29.3	29.6	0.7151	0.99 (0.92–1.1)
Aripiprazole	9.5	7.8	0.0001	1.24 (1.11–1.39)	25.3	23.2	0.0031	1.12 (1.04–1.2)
Any long-acting antipsychotics	7.0	6.1	0.0258	1.15 (1.02–1.31)	20.1	22.1	0.0017	0.89 (0.82–0.96)

ADHD medication is mostly methylphenidate.

### Mortality

During the follow-up (of up to 17 years), 15.4% (*N* = 1387) of men and 10.7% (*N* = 765) of women died. Mortality rate per 100 person-years was 1.58 (95% CI 1∙57–1∙59) for men and 1.03 (95% CI 1.02–1.04) for women. The hazard ratio (HR) for women was 0.65 (CI 0.60–0.71) as compared to men. When adjusted for age and comorbidity, mortality was still significantly lower in women: HR = 0.54 (95% CI: 0.50–0.60). Men had higher incidence rate of death by suicide (2.90 suicides per 1000 person-years, 95% CI 2.89–2∙91 vs. 1.60, 95% CI 1.59–1.61 in women), cardiovascular disease (4.14 per 1000-person-years, 95% CI 4.13–4∙16 vs. 2.65, 95% CI 2.64–2.66 in women) and other causes (7.25, 95% CI 9.23–9.27 vs. 4.22, 4.21–4.24 in women), whereas women had higher cancer mortality rate (1.82, 1.81–1.83 in women vs. 1.49, 1.48–1.50 in men).

## Discussion

We investigated the clinical trajectory of SSD in women and men using the Finnish Hospital Discharge Register maintained by the National Institute of Health and Welfare. Data were also retrieved from the National Prescription Register. To our knowledge, this is the largest cohort describing the course of illness of SSD before and after diagnosis in a gender specific way.

Our incidence gender ratio of 1:1.3 is close to the 1:1.4 of Jongsma et al.^11^ and the gender difference in incidence rates between 1.28 and 1.56 reported for Quebec^12^. The higher incidence in men may partly reflect higher genetic vulnerability and protective effects of estrogens, but higher premorbid substance abuse showed in men is another important contributing factor^13,14^. We found that the vast majority of both women (71%) and men (70%) had been admitted to a psychiatric ward before their first hospitalization for SSD, indicating that most patients were already service users at time of diagnosis. In women, the last hospitalization before the SSD diagnosis was for a mood disorder in 61% (Fig. 1). Most patients already used psychiatric medication before SSD diagnosis, with the early premorbid phase dominated by the use of antidepressant medication and the later premorbid phase dominated by the use of antipsychotics and finally the use of benzodiazepine.

Age of diagnosis of SSD was relatively late in both sexes (34 in men and 38 in women), which may partly be due to the fact that treatment for SSD had already started under a different psychotic diagnosis, as was the case in 55% of women and 54% of men. Our finding that mean age of onset is relatively old in both sexes (i.e., above 30 years of age) aligns with Haukka et al.^15^, who showed that in individuals belonging to the susceptible part of the population, the risk of developing schizophrenia increases with age, at least up to the age of 40. Age at onset peaked around the age of 22 in men, but showed a more plateau-like phase in women, starting after the age of 20 and only slightly decreasing over the years with annual incidences still between 1 and 2% up until the age of 65. We did not see a clear second “post-menopausal” peak, as previously described^16^. Our gender difference in age of onset for SSD was 4 years, which is markedly more than the 1 year observed in meta-analysis^17^. We found that in women, but not in men, there was gap of 4 years between the last hospitalization in the premorbid trajectory and the first admission for SSD. Given the fact that 71% of women later diagnosed with SSD were already service users, screening for SSD among service users, especially in programs for affective disorders may help to shorten diagnostic delay in female patients. After diagnosis, comorbidity remained strongly gendered with more substance abuse in men, but other comorbidities, especially mood disorders, being more frequent in women. Suicide attempts and self-harm was also higher in women both before and after SSD diagnosis. Medication use during the first five years after diagnosis differed for antipsychotics: women slightly more often used antipsychotics than men (OR 1.7), with more prescriptions for quetiapine and aripiprazole and fewer for clozapine, olanzapine and long-acting medication. This suggests that women receive on average less effective types of medication than men. We also found a gender difference for other psychiatric medication: more often antidepressants, mood stabilizers, benzodiazepines and other sedatives for women as compared to men. The higher use of sedatives in women is remarkable, as comorbid substance abuse is lower in women. It could be a by-product of the choice for less sedative antipsychotic regimen in women (i.e. clozapine and olanzapine).

During the follow-up time of ten years, 69.5% of both women and men needed at least one psychiatric re-hospitalization, with slightly more hospitalizations for women and similar mean duration. This suggests that relapse rate, one of the reasons for re-hospitalization is similar among men and women. As these data provide no evidence of a milder course in women, caution should be taken not to underestimate female schizophrenia. The choice for less effective antipsychotics in women (i.e., fewer prescriptions for clozapine and long-acting antipsychotics) may underlie this lack in female advantage for re-hospitalizations. Despite women in this sample being some 4 years older, women had about 50% lower relative risk of death. Suicide and cardiovascular deaths were more common in men, while women had more deaths caused by cancer. Our gender difference in mortality aligns with worldwide meta-analytic data^9^. This large gender difference in mortality may partly result from healthier life style in women (lower nicotine and other substance abuse) and partly from lower mortality rate due to suicide in women. Thus, despite lower rates of suicide attempts, death by suicide is considerably higher in men (almost 3 per 1000 person-years in the unrestricted follow-up of up to 17 years after diagnosis). This gender difference probably results from the more aggressive methods for suicide attempts used by men as compared to women.

Although most of our findings align with previous reports, we provide new insights on two main points. One important new finding is that there seems to be a gap between age 34 (last hospitalization before diagnosis of SSD) and age 38 (first hospitalization for SSD) (Fig. 2), which is suspect for a diagnostic delay in women. The premorbid trajectory of women is less specific and it may be harder to identify SSD in women at an early stage. For example, in women the last hospitalization before diagnosis of schizophrenia is typically for a mood disorder, which may increase the risk of being misdiagnosed as having psychotic depression. Also, higher rates of self-harm and suicide attempts may increase the risk of being misdiagnosed as borderline personality disorder. In Norway, the average time after first presentation until diagnosis of SSD in women was 2.6 years, which is a full year longer than for men^18^, which is probably the same in other countries. Another Norwegian study showed that DUP correlated to severity of depressive symptoms for women, but not for men^19^. It could indicate that the stress of a long DUP generates depressive symptoms per se, but it is also possible that women are more often misdiagnosed as suffering from (psychotic) depression instead of psychosis and that this in turn leads to a delay in treatment. The female premorbid period might be shortened by screening women participating in other treatment programs, especially those for affective disorder for early symptoms of SSD, for example using the Comprehensive Assessment of At Risk Mental States (CAARMS)^20^. As incidence of SSD remains high in women between age 18 and 65, early recognition programs for women should not focus on young adults, but include the full age range. Our second new finding is that women do not have fewer re-hospitalizations than men, neither for psychosis, nor for other psychiatric disorders, which does not support a milder course in women previously reported^21–23^. In line with our finding, a meta-analysis also found largely similar recovery rates for women (12.9%) and men (12.1%)^24^. These findings repudiate the milder course of illness and issue a warning against undertreatment of women with SSD. Pharmacotherapy should certainly take gender into account, as there are sex differences in pharmacokinetics, liver enzymes and renal elimination, which necessitate dose-adjustment for both oral and injectable antipsychotics. Given the sex difference in CYP enzyme activity and dopamine D2 receptor occupancy, women on average need lower dose for olanzapine and clozapine^25^. However, given their equally high risk for re-hospitalization compared to men, women should not be prescribed less effective antipsychotic options. Thus, treatment with clozapine and long-acting antipsychotics should be considered for women just as much as for men. Finally, women with schizophrenia may have a different course, but not necessarily a lighter course. The findings that women have more frequent comorbid depression, more frequent mania, more self-harm and more suicide attempts, together with the higher use of antidepressants, mood stabilizers and sedatives indicate that the female trajectory of schizophrenia is on average more towards the bipolar spectrum as compared to men. Higher prescriptions of sedative drugs in women may reflect higher levels of distress. Suicide attempts and self-harm, clear signs of high levels of distress were higher in women too. Together with the high prevalence of comorbid depression, suffering may actually be higher in women with SSD as compared to men^26^.

The Finnish registers have clear advantages, most importantly; there is no selection bias, as all patients are automatically registered as such and there is hardly any lost to follow-up. In cohort studies and RCTs, patients with suicidality, aggressive incidents, substance abuse and severe comorbidities are often underrepresented, which is not the case in registry studies, providing real-life data. Also, the sample is larger than in RCTs or cohort studies and follow-up time is long.

The fact that diagnoses are clinician-based makes this cohort comparable to clinical practice, which is a clear advantage. However, this also implies that diagnoses may be made based on the stereotype schizophrenia picture, which men resemble more than women do. In addition, the mere fact that patients were female may also have prolonged diagnostic delay, as previous experiments showed that women with similar symptoms as men more often receive a diagnosis other than schizophrenia^18^. We believe that the Finnish health care system is comparable to that of most other European countries, Australia, Canada and Israel, which makes our findings representative for at least part of the world. Potential disadvantage is that not all information is available. For example, details on symptom severity, cognition and dose of medication are lacking. Another disadvantage is that diagnosis is only recorded in combination with hospital admission. As a psychiatric diagnosis is not always coupled to hospital admission, the small part of SSD patients who never were hospitalized was not included in the data on comorbidity and mortality. Previous studies found that in 18% of SSD patients diagnosis and treatment took place in outpatient setting only^4,12^, so we may have presented data on comorbidity and mortality of only some 80% of the true Finnish SSD population. To overcome this problem, we also describe medication use in the period before and after diagnosis, which is registered irrespective of hospital admission. In our data, we found large overlap in comorbidities (i.e., diagnoses coupled to hospitalizations) and medication use (irrespective of hospitalization), which indicates that diagnosis at hospitalization is a reasonable reflection of the total distribution of diagnoses.

In conclusion, gender differences are extensive for comorbidity (both before and after diagnosis), age at diagnosis and mortality, while risk for re-hospitalization and mean number of hospitalizations are not gendered. The suggestion of a diagnostic delay in women urge the use of gendered approaches in early detection programs. The absence of lower re-hospitalization rates in women can be taken as a warning not to undertreat women with SSD. Finally, while the course of schizophrenia is different in women, it is not necessarily lighter than in men.

## Methods

### Study population

The base cohort included all persons hospitalized for a schizophrenia-spectrum disorder (SSD) between 1972 and 2014 in Finland^27^. From the base cohort, all patients newly diagnosed with a schizophrenia-spectrum disorder (SSD), consisting of either schizophrenia or schizo-affective disorder (ICD-10: F20 or F25) with ages between 16–65 years, during 2000–2014 in Finland were included. Persons with SSD were identified from the Hospital Discharge register maintained by the National Institute of Health and Welfare. We restricted analyses to persons diagnosed in their working age (16–65 years). Data for this cohort were also retrieved from the National Prescription register (maintained by Social Insurance Institution, 1995–2017, covering all reimbursed prescription medication dispensings), the National Death Register (dates and causes of death 1972–2017) and the Hospital Discharge Register (1972–2017) which includes all inpatient hospital stays in Finland, recorded for all residents. The year 2000 was chosen to ensure follow-up time of at least five years for medication use before the diagnoses of SSD, since the National Prescription Register starts from year 1995. A longer premorbid period would have been informative, but was not feasible using this database. The research project was approved by the ethics committee of the Finnish National Institute for Health and Welfare. Further permissions were granted by pertinent institutional authorities at the National Institute for Health and Welfare of Finland, the Social Insurance Institution of Finland, and Statistics Finland. As this study uses the Registry data, written informed consent was not needed.

### Hospitalizations before and after diagnosis

Psychiatric hospitalizations in patients later diagnosed with SSD were assessed during five years before diagnosis. The number and durations of hospitalizations for SSD and prevalence of other psychiatric diagnoses was also determined up to ten years after the index diagnosis. Psychiatric diagnoses were defined on ICD-10 (and ICD-9) basis (Table 1 in the supplementary material). Information on diagnoses were collected from Hospital Discharge Register data and these diagnoses are recorded during hospitalization. Therefore, we use psychiatric diagnoses made during hospitalization as a reflection of total diagnoses.

### Medication use

Medication use was assessed during five years before and after diagnosis with SSD. Data on medication use were obtained from the Finnish Prescription Registry, which is not coupled to diagnosis. Specifications of medications assessed are provided in Supplementary Table 2. Medication use was modeled with the PRE2DUP method from purchases into drug use periods as previously described in detail^28^.

### Mortality

Mortality after SSD diagnoses was compared between genders. Persons were censored at the end of study follow-up (December 31, 2017). Causes of death were assessed for broad categories (suicide, cardiovascular disease and cancer).

Figure 1 in Supplementary material provides an overview of study design and outcomes.

### Statistical analysis

Descriptive statistics were calculated as means with standard deviations (SD). When the distributions were highly skewed, we also calculated medians with interquartile ranges (IQR) and percentages. Prevalences of comorbidities and medication use were compared between women and men (men as reference) with logistic regression and reported as Odds ratios (ORs) with 95% confidence intervals (CI) and *p*-values. *p*-values for continuous variables with skewed distributions (age) were calculated with Wilcoxon rank sum test. We used Bonferroni correction for multiple comparisons. Incidence rates were calculated as the number of events divided by person-years. Mortality was assessed with Cox proportional hazard model, with death as an outcome and by censoring to end of follow-up time, and expressed as HRs with 95% CIs.

### Reporting summary

Further information on research design is available in the Nature Research Reporting Summary linked to this article.

## Supplementary information

supplementary material

Reporting Summary

## Data Availability

All data generated or analysed during this study are included in this published article (and its supplementary information files).

## Code availability

Codes used are provided in the supplementary information files.
