# Supplementary material for: The clinical course of schizophrenia in women and men—a nation-wide cohort study
Source: NPJ Schizophr. 2020 May 1;6:12. doi: 10.1038/s41537-020-0102-z (PMC7195359; doi:10.1038/s41537-020-0102-z)
Supplement: Supplementary file 1 — supplementary material [file 41537_2020_102_MOESM1_ESM.pdf]

Supplementary material

**Medication category**	**ATC index**
Antipsychotics	N05A
Antidepressants	N06A
SSRIs	N06AB
Other antidepressants	N06AA, N06AG02, N06AX
Mood stabilizers	N03AF01, N03AG01, N03AX09, N05AN01
Benzodiazepines	N05BA, N05CD, N05CF
Zopiclone and zolpidem	N05CF01, N05CF02
ADHD medication	N06AB
Clozapine	N05AH02
Olanzapine	N05AH03
Quetiapine	N05AH04
Risperidone	N05AX08
Aripiprazole	N05AX12
Any long acting antipsychotic	LAI formulations of N05AB02, N05AB03, N05AC04D, N05AD01, N05AF01, N05AF05, N05AH03, N05AX08, N05AX12, N05AX13

Table 1: Medication categories used with ATC indices.

**Diagnostic category**	**ICD 10**	**ICD 9**
Substance-use related disorders	F10-F19, F55	291, 292, 303, 304, 305
Other psychotic disorders	F21, F22, F23, F28, F29	297, 298
Mood disorders with mania	F30, F31, F34.0	2962-2964, 2967, 3011D
Mood disorders without mania	F32-F39, excl. F34.0	2961
Phobias and anxiety disorders	F40-F41	3000, 3002, 3003
OCD	F42	3003A
PTSD	F43.1	3098X
Dissociative, somatoform and neurasthenic disorders	F44, F45, F48	3078, 3006A, 3008A, 3009X, 3001
Personality disorders	F60-F69	3010A, 3012A, 3017A, 3018D, 3015A, 3014A, 3018C, 3016A, 3018X, 3123A, 3123B, 3123C, 3120A, 3123X, 3025A, 3026A, 3029X, 3028B, 3024A, 3028C, 3022A, 3028D, 3029X, 3026A, 3020A, 3028X, 3015B, 3120A
Eating disorders	F50	3071A, 3075B, 3075E, 3075A
Sleeping disorders	F51	3074
Mental retardation	F70-F79	317-319
Developmental disorders autism spectrum	F80-F89	2990A, 2998A, 3150-3155, 3159
Developmental disorders ADHD spectrum	F90-98	314, 3120A, 3092A, 3138A, 3138X, 3133A, 3132C, 3072B, 3072C, 3072A, 3076A, 3077A, 3075A, 3075C, 3073A, 3070B, 3079X
Suicide/ self-harm	X60–X84, Y10–Y34	E950A, E954A, E955A, E956A, E957A, E959A, E959X, E970A, E972A, E973A, E974A, E979A

Table 2: diagnostic categories used and corresponding codes for ICD 9 and 10.

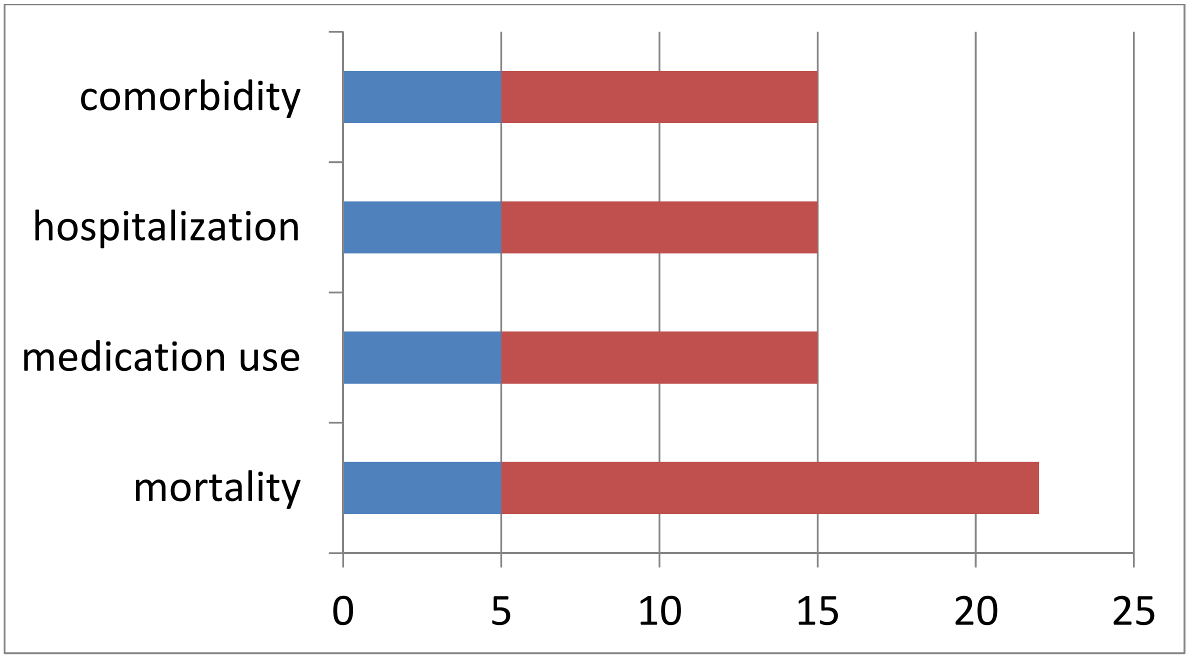

Supplementary Figure 1. Study design and outcomes of interest before and after schizophrenia-spectrum diagnosis.
Time axis in years. Assessment time before first hospitalization for schizophrenia or schizoaffective disorder in blue, assessment time after that diagnosis in red. Medication use was recorded 5 years before and five years after diagnosis. Hospitalization was recorded five years before and up to ten years after diagnosis. Mortality was only recorded after diagnosis with a follow-up period of up to 17 years.

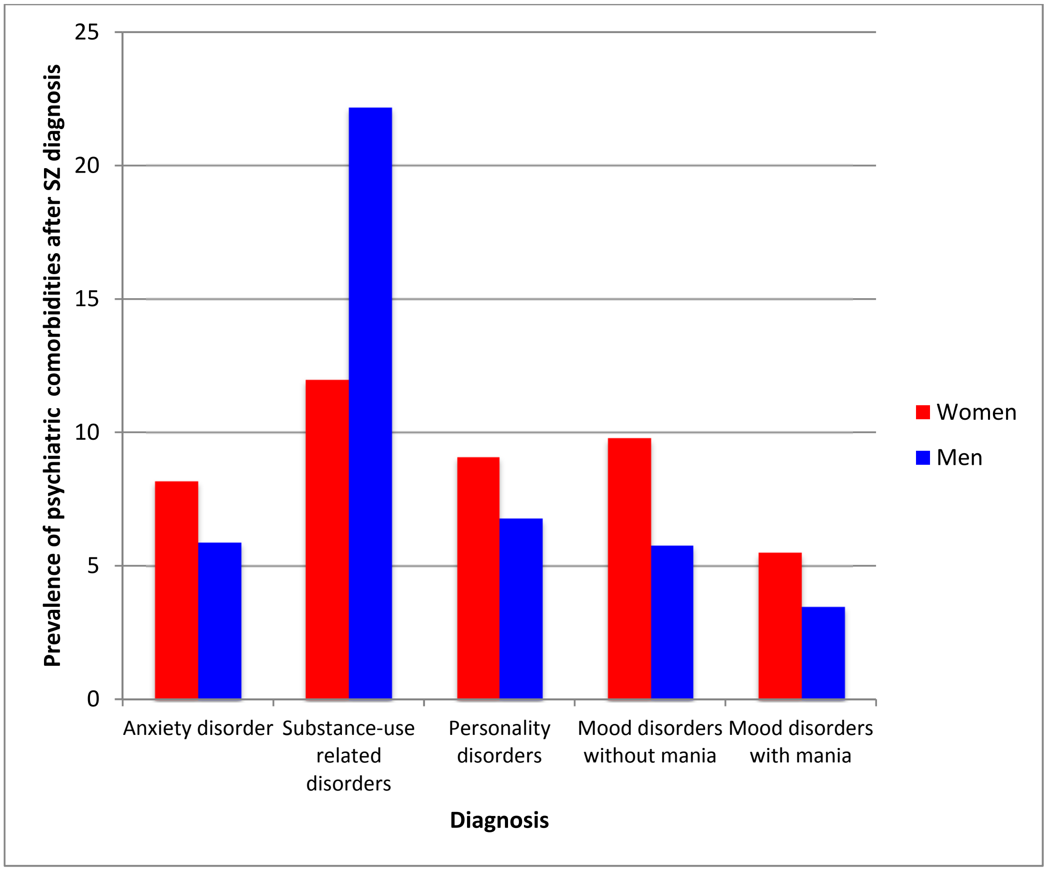

Supplementary Figure 2. Prevalence of psychiatric comorbidities after the schizophrenia-spectrum (SZ) diagnosis categorized by gender (men = 9006, women = 7142).

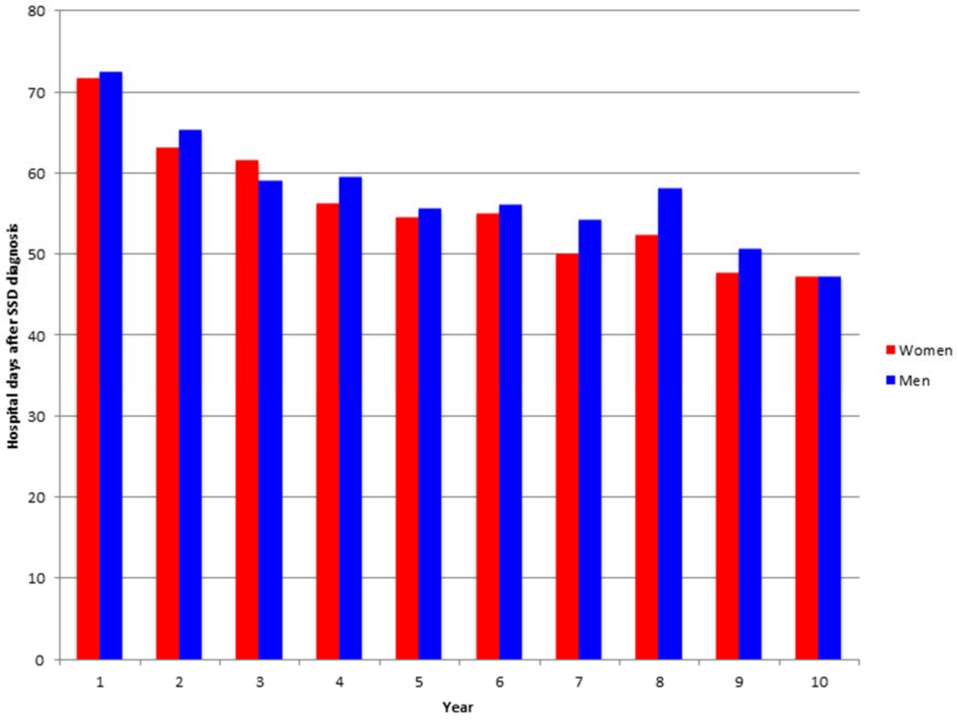

Supplementary Figure 3. Mean number of hospital days for any psychiatric disorder after the diagnosis of schizophrenia-spectrum disease for those who had hospital care days each year (at first year, for 2974 women and 3767 men).

.
